# Activin B promotes endometrial cancer cell migration by down-regulating E-cadherin via SMAD-independent MEK-ERK1/2-SNAIL signaling

**DOI:** 10.18632/oncotarget.9483

**Published:** 2016-05-19

**Authors:** Siyuan Xiong, Christian Klausen, Jung-Chien Cheng, Peter C.K. Leung

**Affiliations:** ^1^ Department of Obstetrics and Gynaecology, Child & Family Research Institute, University of British Columbia, Vancouver, British Columbia V5Z 4H4, Canada

**Keywords:** activin B, E-cadherin, ERK1/2, cell migration, serous endometrial cancer

## Abstract

High-risk type II endometrial cancers account for ~30% of cases but ~75% of deaths due, in part, to their tendency to metastasize. Histopathological studies of type II endometrial cancers (non-endometrioid, mostly serous) suggest overproduction of activin B and down-regulation of E-cadherin, both of which are associated with reduced survival. Our previous studies have shown that activin B increases the migration of type II endometrial cancer cell lines. However, little is known about the relationship between activin B signaling and E-cadherin in endometrial cancer. We now demonstrate that activin B treatment significantly decreases E-cadherin expression in both a time- and concentration-dependent manner in KLE and HEC-50 cell lines. Interestingly, these effects were not inhibited by knockdown of SMAD2, SMAD3 or SMAD4. Rather, the suppressive effects of activin B on E-cadherin were mediated by MEK-ERK1/2-induced production of the transcription factor SNAIL. Importantly, activin B-induced cell migration was inhibited by forced-expression of E-cadherin or pre-treatment with the activin/TGF-β type I receptor inhibitor SB431542 or the MEK inhibitor U0126. We have identified a novel SMAD-independent pathway linking enhanced activin B signaling to reduced E-cadherin expression and increased migration in type II endometrial cancer.

## INTRODUCTION

Endometrial cancer is the second most lethal gynecological malignancy in North America. While the mortality rates of many cancers have been effectively reduced, the number of deaths due to endometrial cancer continues to rise [1]. Large, population-based studies of the Surveillance, Epidemiology, and End Results (SEER) database suggest this phenomenon can likely be attributed to the increased incidence of advanced-stage tumors and high risk histologies [2]. Endometrial cancers have traditionally been classified into type I or type II tumors as defined by Bokhman [3]. Whereas type I tumors are estrogen-dependent, non-metastatic, and associated with favorable prognosis, type II tumors tend to be estrogen-independent, highly invasive and more lethal. Type I endometrial cancers are generally low-grade endometrioid tumors whereas type II endometrial cancers are mostly of serous, clear cell or high-grade endometrioid histology [4, 5]. Type II endometrial cancers account for ~30% of cases but ~75% of deaths due, in part, to their tendency to metastasize. In particular, serous endometrial carcinomas account for ~40% of all endometrial cancer deaths and are extremely aggressive, with relapse rates as high as 50% and 5-year overall survival rates as low as 18-27% [4, 6, 7]. Thus, a deeper understanding of the molecular pathways involved in the invasion and metastatic spread of type II endometrial cancers is needed in order to develop new therapeutic approaches with the potential to improve patient outcomes.

Activins belong to the transforming growth factor-β (TGF-β) superfamily of cytokines, which includes TGF-βs, activins, nodal, inhibins, growth differentiation factors (GDFs), and bone morphogenetic proteins (BMPs). Activins are homo- or hetero-dimers of inhibin β subunits and the primary isoforms are activin A (βAβA), activin AB (βAβB) and activin B (βBβB). Activins bind to type II transmembrane serine-threonine kinase receptors (ACVR2A or ACVR2B) which, in turn, activate type I receptors (ACVR1B) that phosphorylate and activate canonical SMAD signaling pathways. Activins have also been shown to signal in a SMAD-independent manner via the phosphoinositide 3-kinase (PI3K)/AKT and mitogen-activated protein kinase (MAPK) pathways [8]. Activins are overexpressed and correlated with poor prognosis and survival in a variety of human malignancies [8]. More importantly, inhibin β subunit expression, activin secretion and activin receptor expression have been demonstrated in neoplastic endometrial tissues and/or endometrial cancer cell lines [9–11]. Histopathological studies of inhibin βA and βB subunit expression in endometrial cancers of endometrioid histology failed to show any association with survival [12, 13]. However in a study of 41 non-endometrioid tumors, of which 70% were serous, positive immunostaining for inhibin βB was associated with reduced cause specific survival and trends towards reduced progression free and overall survival [14]. Similarly, our recent analysis of serous endometrial cancers from The Cancer Genome Atlas (TCGA) ([15]; n=53) showed that elevated inhibin βB mRNA levels are associated with reduced disease free survival and a trend towards reduced overall survival [16]. These studies support the hypothesis that activin B (βBβB) signaling may be linked to poor survival in type II endometrial cancer. Indeed, we have shown that activin B treatment can promote the adhesion, migration and invasion of type II endometrial cancer cells by up-regulating integrin β3 in a SMAD-dependent manner [16].

Cancer cell metastasis is also closely associated with epithelial-mesenchymal transition (EMT) which is characterized by the down-regulation of E-cadherin [17]. E-cadherin is a calcium dependent trans-membrane glycoprotein that plays a key role in the formation of adherens junctions between epithelial cells. E-cadherin expression is regulated by a group of transcription factors associated with the process of EMT, such as SNAIL, SLUG, TWIST and ZEB1 [18]. Reduced E-cadherin expression or loss of function are correlated with metastasis and adverse clinical outcomes in several types of cancer [19–23], whereas overexpression markedly impairs cancer cell invasiveness [21, 22, 24, 25]. In endometrial cancer, decreased E-cadherin is associated with adverse clinicopathological factors and poor survival. In addition, several studies have reported lower levels of E-cadherin in type II compared to type I endometrial cancers [26–30], which may explain the more aggressive behavior of type II tumors. However, the exact roles of E-cadherin in type II endometrial cancer and the mechanisms responsible for its down-regulation remain poorly understood. Activin A has been shown to promote the migration of different cell types concomitant with E-cadherin down-regulation [31–33]. In the current study, we investigated the effects of activin B on E-cadherin expression in type II endometrial cancer cells. We demonstrate that activin B suppresses the expression of E-cadherin by up-regulating SNAIL via MEK-ERK1/2 signaling, thereby enhancing KLE and HEC-50 cell migration.

## RESULTS

### Activin B down-regulates E-cadherin in human endometrial cancer cells

**Figure 1:**
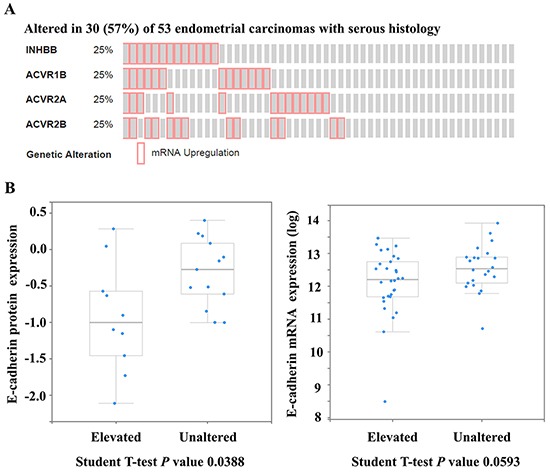
Enhanced activin B signaling may contribute to the down-regulation of E-cadherin in serous endometrial cancers The cBioPortal for Cancer Genomics was used to query serous endometrial carcinomas from The Cancer Genome Atlas (n=53) for up-regulation (upper quartile) of at least one component of the activin B ligand-receptor gene set, including inhibin βB (INHBB), type I receptor ACVR1B, and type II receptors ACVR2A and ACVR2B. **A.** OncoPrint showing cases with elevated mRNA levels of INHBB, ACVR1B, ACVR2A or ACVR2B across all 53 serous endometrial carcinomas. **B.** Enrichment analysis comparing E-cadherin protein (*left*) and mRNA (*right*) levels between unaltered samples and those with INHBB, ACVR1B, ACVR2A or ACVR2B mRNA levels in the upper quartile (elevated). Expression levels of E-cadherin protein (from reverse-phase protein array) and mRNA (from RNA-Seq V2 RSEM) are displayed as boxplots with a *P* value from a Student T-test.

To investigate the relationship between activin B signaling and E-cadherin in serous endometrial carcinoma (TCGA; n=53; [15]), we used the cBioPortal for Cancer Genomics to perform enrichment analysis comparing E-cadherin levels between unaltered samples and those with elevation of at least one component of the activin B ligand-receptor gene set, including inhibin βB (INHBB), type I receptor ACVR1B, and type II receptors ACVR2A and ACVR2B (Figure 1). Serous tumors with INHBB, ACVR1B, ACVR2A or ACVR2B mRNA levels in the upper quartile displayed reduced levels of E-cadherin protein (*P* = 0.039) and a trend towards reduced levels of E-cadherin mRNA (*P* = 0.059). These findings suggest that enhanced activin B signaling may contribute to the down-regulation of E-cadherin in type II serous endometrial cancer.

**Figure 2:**
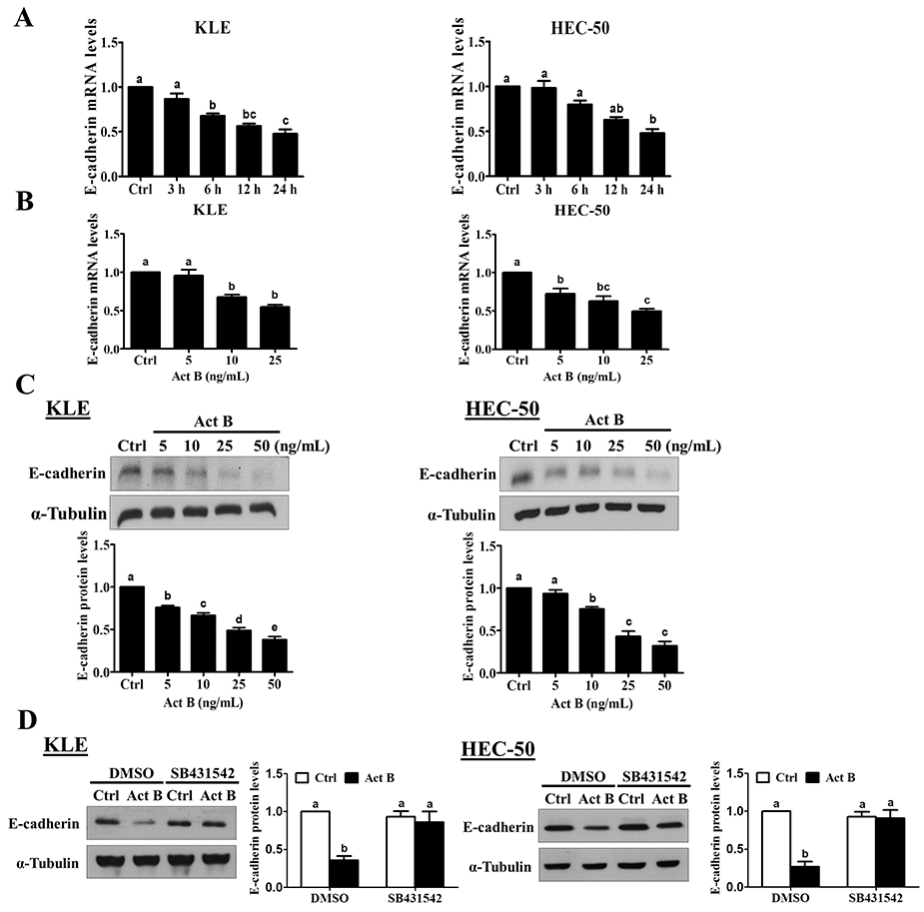
Activin B down-regulates E-cadherin expression in human endometrial cancer cells **A.** KLE and HEC-50 cells were treated for varying times without (Ctrl; time-matched controls displayed as single bar) or with 50 ng/mL activin B and E-cadherin mRNA levels were examined by RT-qPCR. **B.** and **C.** KLE and HEC-50 cells were treated for 24 h with increasing concentrations of activin B (5, 10, 25 or 50 ng/mL) and E-cadherin mRNA (B) and protein (C) levels were measured by RT-qPCR and Western blot, respectively. **D.** KLE and HEC-50 cells were pre-treated with vehicle control (DMSO) or SB431542 (10 μM) for 1 h and then treated with or without 50 ng/mL activin B (Act B) for 24 h. Protein levels of E-cadherin were examined by Western blot. Results are expressed as the mean ± SEM of at least three independent experiments and values without common letters are significantly different (*P* < 0.05).

To examine the effect of activin B on E-cadherin expression, we treated KLE and HEC-50 type II human endometrial cancer cell lines with 50 ng/mL activin B for different periods of time (3, 6, 12 or 24 h). As shown in Figure 2A, treatment with activin B down-regulated E-cadherin mRNA levels in a time-dependent manner in both KLE and HEC-50 cells, with maximal effects observed 24 h after activin B treatment. Next, we measured E-cadherin mRNA and protein levels following treatment for 24 h with increasing concentrations of activin B (5, 10, 25 or 50 ng/mL). As shown in Figure 2B and 2C, treatment with activin B down-regulated E-cadherin in a concentration-dependent manner, with effects observed at concentrations as low as 5-10 ng/mL. Furthermore, these reductions in E-cadherin protein were abolished by pre-treatment with the activin/TGF-β type I receptor inhibitor SB431542 (Figure 2D).

### SMAD signaling is not required for activin B-induced down-regulation of E-cadherin

**Figure 3:**
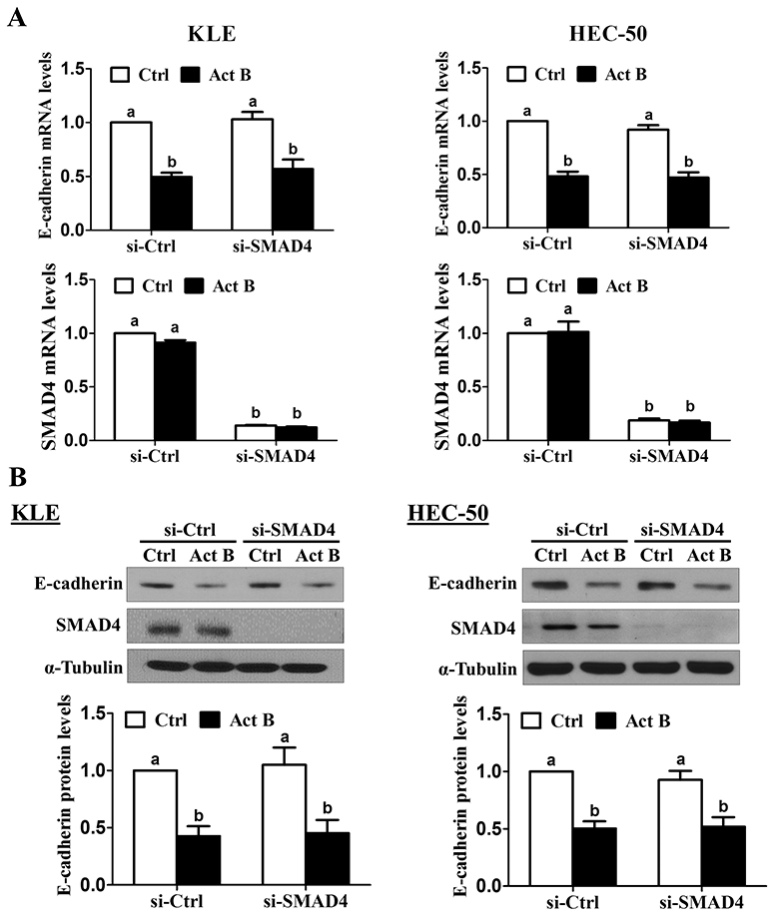
SMAD4 is not required for the down-regulation of E-cadherin by activin B KLE and HEC-50 cells were transfected for 48 h with 20 nM control siRNA (si-Ctrl) or SMAD4 siRNA (si-SMAD4) and then treated without (Ctrl) or with 50 ng/mL activin B (Act B) for 24 h. E-cadherin and SMAD4 mRNA **A.** and protein **B.** levels were measured by RT-qPCR and Western blot, respectively. Results are expressed as the mean ± SEM of at least three independent experiments and values without common letters are significantly different (*P* < 0.05).

**Figure 4:**
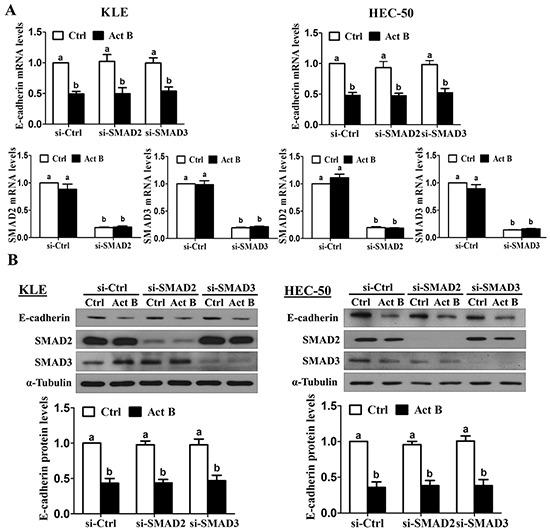
SMAD2 and SMAD3 are not required for activin B-induced down-regulation of E-cadherin KLE and HEC-50 cells were transfected for 48 h with 20 nM control siRNA (si-Ctrl), SMAD2 siRNA (si-SMAD2) or SMAD3 siRNA (si-SMAD3) and then treated without (Ctrl) or with 50 ng/mL activin B (Act B) for 24 h. E-cadherin, SMAD2 and SMAD3 mRNA **A.** and protein **B.** levels were measured by RT-qPCR and Western blot, respectively. Results are expressed as the mean ± SEM of at least three independent experiments and values without common letters are significantly different (*P* < 0.05).

We have previously shown that treatment with activin B phosphorylates/activates SMAD2 and SMAD3 in type II human endometrial cancer cells [16]. To examine the involvement of SMAD signaling in activin B-induced down-regulation of E-cadherin, KLE and HEC-50 cells were transfected with siRNA targeting common SMAD4 prior to treatment with activin B. As shown in Figure 3A, despite reducing SMAD4 mRNA levels by more than 80%, pre-treatment with SMAD4 siRNA did not alter the inhibitory effects of activin B on E-cadherin mRNA levels in either cell line. Similarly, Western blot analysis showed that the suppressive effects of activin B on E-cadherin protein levels were not affected by SMAD4 knockdown (Figure 3B). Next, we used specific siRNAs targeting SMAD2 or SMAD3 to further confirm that SMAD signaling is not required for the down-regulation of E-cadherin by activin B in KLE and HEC-50 cells. Whereas transfection with SMAD2 or SMAD3 siRNA significantly reduced their respective protein and mRNA levels by more than 75%, neither siRNA altered the inhibitory effects of activin B on E-cadherin mRNA and protein levels (Figure 4).

### MEK-ERK1/2 signaling is required for the down-regulation of E-cadherin by activin B

**Figure 5:**
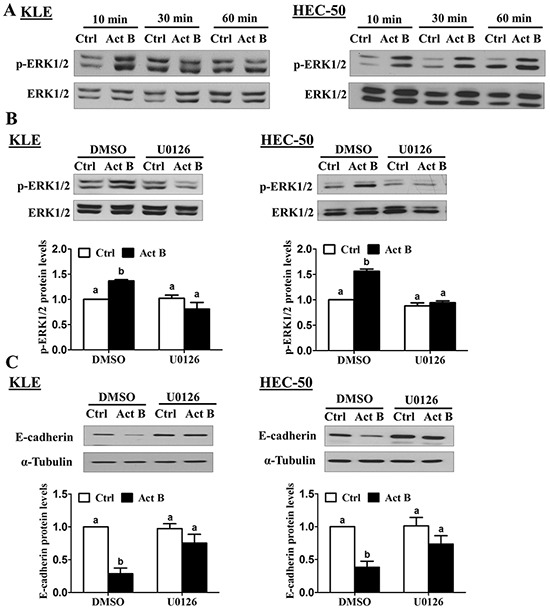
MEK-ERK1/2 signaling is required for the down-regulation of E-cadherin by activin B **A.** KLE and HEC-50 cells were treated without (Ctrl) or with 50 ng/mL activin B (Act B) for 10, 30 or 60 min and Western blot was used to examine the levels of phosphorylated ERK1/2 (p-ERK1/2) in relation to total levels of ERK1/2. **B.** KLE and HEC-50 cells were pre-treated with vehicle control (DMSO) or U0126 (10 μM) for 1 h and then treated with or without 50 ng/mL activin B for 10 min. Western blot was used to measure ERK1/2 phosphorylation. **C.** KLE and HEC-50 cells were pre-treated with or without U0126 (10 μM) for 1 h and then treated with or without 50 ng/mL activin B for 24 h. Protein levels of E-cadherin were examined by Western blot. Results are expressed as the mean ± SEM of at least three independent experiments and values without common letters are significantly different (*P* < 0.05).

Since the effects of activin B on E-cadherin were not mediated by canonical SMAD signaling, we next investigated whether MEK-ERK1/2, PI3K/AKT or p38 MAPK signaling might be involved. To examine the activation of these pathways, we treated KLE and HEC-50 cells with activin B and used Western blot to measure the levels of phosphorylated ERK1/2, AKT and p38 MAPK in relation to their total levels. Whereas treatment with activin B induced the phosphorylation of ERK1/2 in both cell lines after 10 min, ERK1/2 activation was more prolonged in HEC-50 cells (Figure 5A). In contrast, activin B treatment did not alter the phosphorylation of AKT or p38 MAPK at any of the time-points examined (10, 30 or 60 min; Supplementary Figure S1). We then used the MEK inhibitor U0126 to determine whether MEK-ERK1/2 signaling is required for the effects of activin B on E-cadherin in KLE and HEC-50 cells. Pre-treatment with U0126 inhibited both the induction of ERK1/2 phosphorylation (Figure 5B) and the down-regulation of E-cadherin (Figure 5C) by activin B.

### Activin B down-regulates E-cadherin via MEK-ERK1/2-induced up-regulation of SNAIL

**Figure 6:**
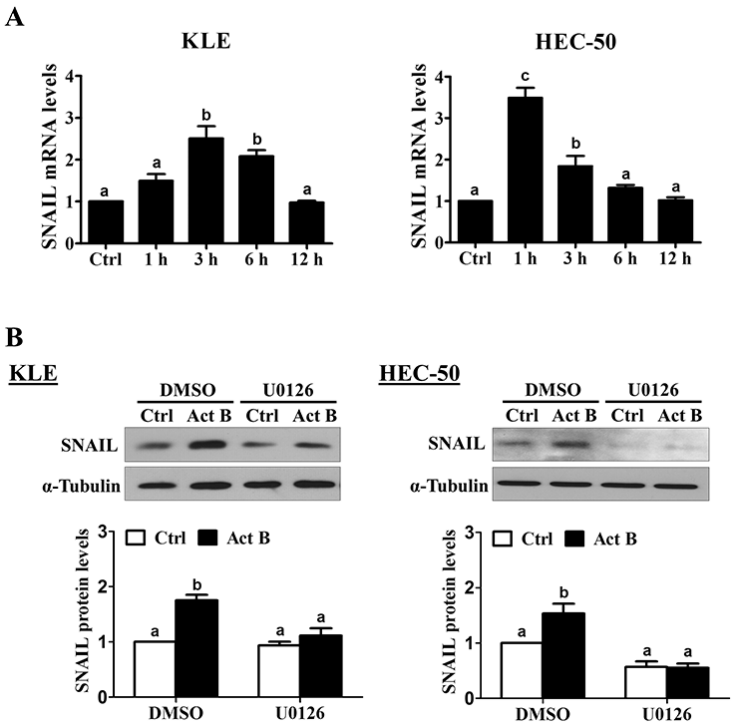
Activin B up-regulates SNAIL via MEK-ERK1/2 signaling **A.** KLE and HEC-50 cells were treated for varying times without (Ctrl; time-matched controls displayed as single bar) or with 50 ng/mL activin B and SNAIL mRNA levels were examined by RT-qPCR. **B.** KLE and HEC-50 cells were pre-treated with vehicle control (DMSO) or U0126 (10 μM) for 1 h and then treated with or without 50 ng/mL activin B (Act B) for 3 h. Protein levels of SNAIL were examined by Western blot. Results are expressed as the mean ± SEM of at least three independent experiments and values without common letters are significantly different (*P* < 0.05).

Next, we examined the effects of activin B on a set of EMT-related transcription factors previously linked to the down-regulation of E-cadherin (SNAIL, SLUG, TWIST and ZEB1 [18]). Treatment of KLE cells with activin B up-regulated SNAIL mRNA levels at 3 and 6 h, whereas significant increases were observed at 1 and 3 h in HEC-50 cells (Figure 6A). In contrast, treatment with activin B did not significantly affect the mRNA levels of SLUG, TWIST or ZEB1 at any of the time-points examined in either cell line (1, 3, 6 or 12 h; Supplementary Figure S2). Western blot results showed that treatment with activin B for 3 h increased SNAIL protein levels in both KLE and HEC-50 cells (Figure 6B). In addition, inhibition of MEK-ERK1/2 signaling by pre-treatment with U0126 abolished the up-regulation of SNAIL protein levels by activin B (Figure 6B).

**Figure 7:**
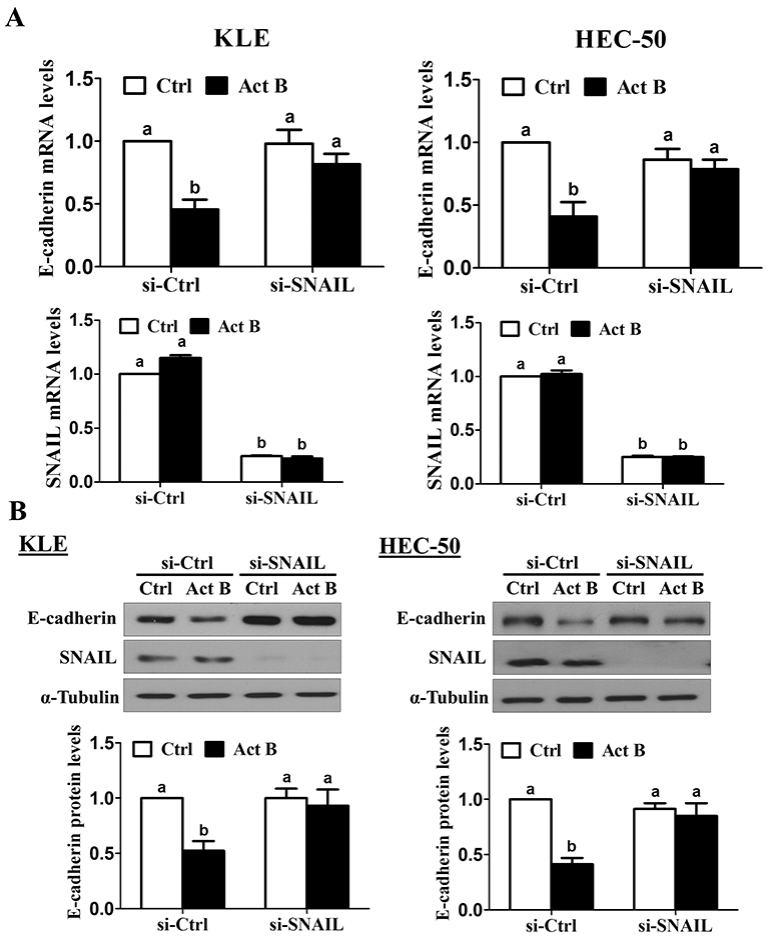
SNAIL is required for the down-regulation of E-cadherin by activin B KLE and HEC-50 cells were transfected for 48 h with 20 nM control siRNA (si-Ctrl) or SNAIL siRNA (si-SNAIL) and then treated without (Ctrl) or with 50 ng/mL activin B (Act B) for 24 h. E-cadherin and SNAIL mRNA **A.** and protein **B.** levels were measured by RT-qPCR and Western blot, respectively. Results are expressed as the mean ± SEM of at least three independent experiments and values without common letters are significantly different (*P* < 0.05).

SNAIL knockdown was used to investigate its involvement in activin B-induced down-regulation of E-cadherin. As shown in Figure 7A, pre-treatment for 48 h with SNAIL siRNA significantly reduced SNAIL mRNA levels and abolished the inhibitory effects of activin B (50 ng/mL, 24 h) on E-cadherin mRNA levels in both KLE and HEC-50 cells. Similarly, knockdown of SNAIL suppressed endogenous SNAIL protein levels and completely blocked activin-B-induced down-regulation of E-cadherin protein (Figure 7B).

### Down-regulation of E-cadherin is required for activin B-induced cell migration

**Figure 8:**
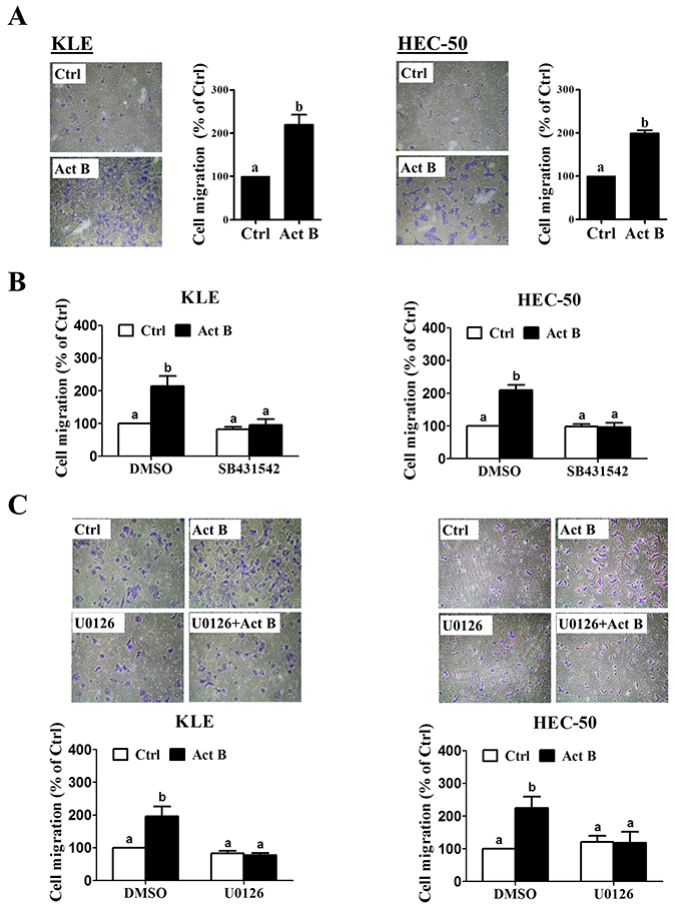
MEK-ERK1/2 signaling is required for activin B-induced cell migration **A.** KLE and HEC-50 cells were treated without (Ctrl) or with 50 ng/mL activin B (Act B) for 24 h and then seeded in transwell inserts for migration assay. For each cell line, *left panels* show representative photomicrographs of migrating cells, while *right anels* show summarized quantitative results. **B.** Migration assays were performed with KLE and HEC-50 cells following pre-treatment with vehicle control (DMSO) or SB431542 (10 μM) for 1 h prior to treatment with or without 50 ng/mL activin B for 24 h. **C.** Migration assays were performed with KLE and HEC-50 cells following pre-treatment with vehicle control (DMSO) or U0126 (10 μM) (C) for 1 h prior to treatment with or without 50 ng/mL activin B for 24 h. For each cell line, upper panels show representative photomicrographs, while lower panels show summarized quantitative results. Results are expressed as the mean ± SEM of at least three independent experiments and values without common letters are significantly different (P < 0.05).

**Figure 9:**
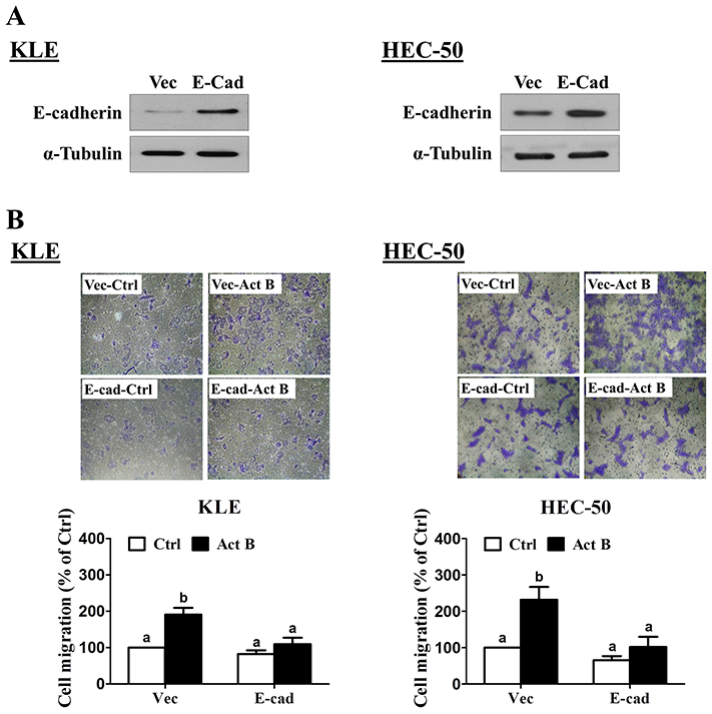
Forced-expression of E-cadherin inhibits activin B-induced cell migration **A.** KLE and HEC-50 cells were transfected for 48 h with control vector (Vec) or vector encoding full-length human E-cadherin (E-cad). Protein levels of E-cadherin were examined by Western blot. **B.** Transwell migration assays were performed with KLE and HEC-50 cells following transfection for 48 h with control vector or E-cadherin vector prior to treatment without (Ctrl) or with 50 ng/mL activin B for 24 h. For each cell line, *upper panels* show representative photomicrographs, while *lower panels* show summarized quantitative results. Results are expressed as the mean ± SEM of at least three independent experiments and values without common letters are significantly different (*P* < 0.05).

Transwell migration assay results confirmed the pro-migratory effects of activin B on both KLE and HEC-50 cells (Figure 8A). Furthermore, these effects of activin B were abolished by inhibition of activin/TGF-β type I receptors (SB431542; Figure 8B) or MEK-ERK1/2 signaling (U0126; Figure 8C). Next, we used transient overexpression of full-length human E-cadherin to further investigate the role of E-cadherin in activin B-induced cell migration. Western blot analysis confirmed the increased production of E-cadherin in KLE and HEC-50 cells transfected with the E-cadherin vector (Figure 9A). Importantly, migration assay results showed that forced-expression of E-cadherin attenuated activin B-increased cell migration in both KLE and HEC-50 cells (Figure 9B).

## DISCUSSION

Like TGF-β1, activins are thought to play both positive and negative roles in tumor development and progression [34]. Nevertheless, blocking the activin/TGF-β pathway has been shown to suppress multiple organ metastases in several types of cancer [35–37]. We have previously shown that activin B enhances the adhesion, migration and invasion of type II endometrial cancer cells in a SMAD-dependent manner [16]. We now describe a novel SMAD-independent pathway contributing to the pro-migratory effects of activin B on type II endometrial cancer cells. These findings could be clinically relevant to type II endometrial cancer, especially the serous subtype, because these cancers are frequently associated with invasion of lymphatic and vascular spaces, lymph node metastases, involvement of other intra-peritoneal structures, and distant recurrences [6, 38, 39]. Characterization of the molecular mechanisms contributing to cell motility and invasiveness could help identify novel therapeutic targets for this most lethal type of endometrial cancer.

Studies suggest that EMT may contribute to endometrial cancer metastasis, in particular the loss of E-cadherin and the activation of transcription factors involved in its repression. Indeed, reduced E-cadherin expression is associated with advanced stage [30, 40], poor differentiation [30, 41], deep myometrial invasion [29, 40, 41], lymph node metastasis [26], and extra-pelvic recurrence [42] in endometrial cancer. More importantly, E-cadherin expression is inversely correlated with survival in endometrial cancer [26, 28, 29, 40, 42, 43]. Interestingly, the expression of E-cadherin is reduced in type II compared to type I endometrial carcinoma, suggesting its loss could contribute to the aggressive behavior of type II endometrial cancers [26–30]. Though the relationship between TGF-β1 and EMT has been well-studied in a variety of cancers [8], much less is known about the roles of activins, especially with respect to the regulation of E-cadherin. Moreover, most studies have examined only the effects of activin A [31–33], despite increasing evidence suggesting that activin isoforms could function differently depending on the cellular context [44]. To date, only one study in clear cell renal cell carcinoma has described the effects of activin B on cell invasion, however activin B did not alter the expression of E-cadherin or its related transcription factors in these cells [45]. We report for the first time that activin B down-regulates E-cadherin in type II endometrial cancer cells, and that forced-expression of E-cadherin reverses activin B-induced cell migration. These cellular effects are consistent with our TCGA analysis showing reduced E-cadherin levels in serous endometrial cancers with elevation of at least one component of the activin B ligand-receptor gene set. Interestingly, no significant differences in E-cadherin protein (*P* = 0.24) or mRNA (*P* = 0.61) levels were observed when similar enrichment analyses were performed with endometrioid endometrial cancers (TCGA; n=307; INHBB, ACVR1B, ACVR2A or ACVR2B mRNA levels in the upper quartile). Moreover, in contrast to serous tumors, where increased inhibin βB immunostaining or mRNA levels are associated with reduced survival [14, 16], neither inhibin βA nor βB expression levels are associated with survival in endometrioid endometrial carcinomas [12, 13]. These findings suggest the relationship between activin B signaling and E-cadherin may be specific to serous endometrial cancer, and may contribute to its aggressive behavior. Our findings suggest that further investigations of activin B signaling or E-cadherin as prognostic biomarkers or therapeutic targets in type II endometrial carcinoma are warranted.

SNAIL is a well-known transcriptional repressor of E-cadherin that is overexpressed in multiple human cancers [46]. Down-regulation of SNAIL attenuates tumor growth and invasiveness in animal models, and its expression is linked to poor prognosis [47]. In endometrial cancer, elevated SNAIL expression is related to advanced stage, myometrial invasion and lymph node metastasis [29, 48]. Interestingly, non-endometrioid tumors appear to have higher rates of nuclear SNAIL expression than endometrioid tumors [29], which may contribute to their metastatic propensity. Up-stream regulators of SNAIL expression in endometrial cancer are not well-defined; however, our study shows that SNAIL can be up-regulated by activin B-induced MEK-ERK1/2 signaling in type II endometrial cancer cells. Moreover, we show that SNAIL is required for activin-B induced down-regulation of E-cadherin. Our findings are consistent with a previous report showing SNAIL up-regulation via ERK1/2-mediated activation of AP-1 transcription factor [47]. ERK signaling also mediates epidermal growth factor-induced SNAIL up-regulation and subsequent cadherin switching and cell invasion in serous borderline ovarian tumor cells [49]. However, the regulation of SNAIL is cell context-dependent as its expression is also governed by SMADs [50] and PI3K-AKT signaling [47].

Activation of SMAD-independent pathways, including MAPK signaling, is well-described for TGF-β [51, 52]. These pathways exert their own independent functions however they can also compliment or antagonize canonical SMAD-dependent signaling [51]. On the other hand, far fewer studies have described SMAD-independent signaling pathways activated by activins. Given that TGF-βs and activins utilize distinct sets of type I and type II receptors, they may each activate a unique complement of SMAD-independent signaling pathways which could result in different functional consequences [8]. Several studies have demonstrated that activins can induce MEK-ERK1/2 signaling, and our finding that MEK inhibition blocks activin A-induced endometrial cancer cell migration is consistent with previous studies in mesothelioma and mesenchymal stem cells [53–55]. However unlike the present study, those previous studies did not rule out SMAD-dependent actions/crosstalk. This is important because multiple MAPKs, including ERK1/2, can regulate the functions of SMAD2 and SMAD3 by phosphorylating a linker region that is not phosphorylated by type I/II receptors [56]. Thus, experimental approaches traditionally thought to address only SMAD-independent signaling (e.g. MAPK inhibitors) have the potential to alter SMAD-dependent actions as well. We used knockdown of SMAD2, SMAD3 or SMAD4 to show that the suppressive effects of activin B on E-cadherin expression are not mediated by canonical SMAD signaling. Interestingly, we have previously demonstrated that both SMAD2 and SMAD3 are required for activin B-induced integrin β3 up-regulation [16]. Together, our studies suggest that type II endometrial cancer cell migration/invasion involves both MEK-ERK1/2-SNAIL-mediated E-cadherin down-regulation and SMAD2/3-SMAD4-mediated integrin β3 up-regulation. In addition to regulating cell migration, MEK-ERK1/2 signaling can promote endometrial cancer cell proliferation [57, 58]. However, a recent phase II study of the MEK inhibitor Selumetinib demonstrated only limited single-agent activity in endometrial cancer [59]. These results could be explained by the presence of other oncogenic signaling pathways, such as PI3K/AKT, Wnt/β-catenin, SMAD, epidermal growth factor receptor/HER2 etc. [60]. In this context, therapeutic approaches targeting multiple pathways may yield improved activity over single-agent treatments. For example, phase II studies are currently underway to assess the efficacy of MEK inhibition (Trametinib) alone or in combination with AKT inhibition (GSK2141795) in endometrial cancer (NCT01935973). Our results suggest that approaches aimed at inhibiting activin receptor activity could impact multiple downstream pathways and warrant further investigation in type II endometrial cancer. Presently, phase I studies with an ACVR2B Fc fusion protein (STM 434) in combination with liposomal doxorubicin are underway in patients with advanced tumors, including endometrial cancer (NCT02262455).

Our study provides important insights into the SMAD-independent actions of activin B in type II endometrial cancer cells. In summary, we demonstrate that activin B induces the activation of MEK-ERK1/2 signaling which stimulates the production of SNAIL. This up-regulation of SNAIL is required for the down-regulation of E-cadherin which is necessary for activin B-induced cell migration. Therapeutic approaches targeting the molecular mechanisms contributing to invasion/metastasis have the potential to significantly improve the clinical outcomes of patients with type II endometrial cancer.

## MATERIALS AND METHODS

### Cell culture

The KLE human endometrial cancer cell line was purchased from the American Type Culture Collection (Manassas, VA). The HEC-50 human endometrial cancer cell line was obtained from the OVCARE Cell Bank (Vancouver, BC). Both cell lines were cultured in DMEM/nutrient mixture F-12 Ham (DMEM/F12; Gibco, Life Technologies, Burlington, ON) supplemented with 100 U/mL penicillin (Gibco, Life Technologies), 100 μg/mL streptomycin (Gibco Life Technologies), and 10% (vol/vol) fetal bovine serum (FBS; Hyclone Laboratories, Logan, UT). Cultures were maintained at 37°C in a humidified atmosphere of 5% CO_2_ in air.

### Antibodies and reagents

The following rabbit polyclonal antibodies were obtained from Cell Signaling Technology (Danvers, MA): human SMAD4 (#9515), human phospho-p44/42 MAPK (ERK1/2, Thr202/Tyr204; #9101), rat p44/42 MAPK (ERK1/2; #9102), mouse phospho-AKT (Ser473; #9271), mouse AKT (#9272), human phospho-p38 MAPK (Thr180/Tyr182; #9211), and human p38 MAPK (#9212). The following rabbit monoclonal antibodies were obtained from Cell Signaling Technology: human phospho-SMAD2 (Ser465/467; 138D4), human phospho-SMAD3 (Ser423/425; C25A9), and human SMAD3 (C67H9). The mouse monoclonal antibodies used were: human SMAD2 (L16D3, Cell Signaling Technology), human E-cadherin (#610404, BD Biosciences, Mississauga, ON), human SNAIL (#3895, Cell Signaling Technology) and sea urchin α-tubulin (B-5-1-2, Santa Cruz Biotechnology, Dallas, TX). Horseradish peroxidase-conjugated goat anti-mouse IgG and goat anti-rabbit IgG were obtained from Bio-Rad Laboratories (Mississauga, ON). SB431542 was purchased from Sigma-Aldrich (Oakville, ON). U0126 was obtained from Calbiochem (San Diego, CA). Recombinant human activin B was obtained from R&D Systems (Minneapolis, MN).

### Transwell migration assay

Cell culture inserts (24-well, pore size 8 μm; BD Biosciences) were seeded with 1 × 10^5^ cells in 250 μL of medium supplemented with 0.1% FBS. Medium with 10% FBS (750 μL) was added to the lower chamber and served as a chemotactic agent. After incubation for 24 h, non-migrating cells were removed from the upper side of the membrane, and the cells on the lower side of the membrane were fixed with cold methanol and air dried. Cells were stained with Crystal Violet and counted using a light microscope. Each individual experiment was performed with triplicate inserts and five microscopic fields were counted per insert.

### Reverse transcription-quantitative real-time PCR (RT-qPCR)

Total RNA was extracted using TRIzol reagent (Invitrogen, Life Technologies, Burlington, ON) in accordance with the manufacturer's instructions. Reverse transcription was performed with 2 mg RNA, random primers and M-MLV reverse transcriptase (Promega, Madison, WI). Each 20 μl SYBR Green RT-qPCR reaction contained 1×SYBR Green PCR Master Mix (Applied Biosystems), 12 ng cDNA and 150 nM of each specific primer. The primers used were: E-cadherin (CDH1), 5′-ACA GCC CCG CCT TAT GAT T-3′ (forward) and 5′-TCG GAA CCG CTT CCT TCA-3′ (reverse); SNAIL (SNAI1), 5′-CCC CAA TCG GAA GCC TAA CT-3′ (forward) and 5′-GCT GGA AGG TAA ACT CTG GAT TAG A-3′ (reverse); SLUG (SNAI2), 5′-TTC GGA CCC ACA CAT TAC CT-3′ (forward) and 5′-GCA GTG AGG GCA AGA AAA AG-3′ (reverse); TWIST (TWIST1), 5′-GGA GTC CGC AGT CTT ACG AG-3′ (forward) and 5′-TCT GGA GGA CCT GGT AGA GG-3′ (reverse); ZEB1, 5′-GCA CCT GAA GAG GAC CAG AG-3′ (forward) and 5′-TGC ATC TGG TGT TCC ATT TT-3′ (reverse); and GAPDH, 5′-GAG TCA ACG GAT TTG GTC GT-3′ (forward) and 5′- GAC AAG CTT CCC GTT CTC AG-3′ (reverse). RT-qPCR was performed using an Applied Biosystems 7300 Real-Time PCR System equipped with 96-well optical reaction plates. The specificity of each assay was validated by melting curve analysis and agarose gel electrophoresis of the PCR products. Assay performance was validated by assessing amplification efficiencies by means of calibration curves, and ensuring that the plot of log input amount versus ΔCq has a slope with an absolute value <0.1. At least three separate experiments were performed and each sample was assayed in triplicate. A mean value of the triplicates was used for the determination of relative mRNA levels by the comparative Cq method with GAPDH as the reference gene and using the formula 2^−ΔΔCq^.

### Western blot

Cells were lysed in ice cold lysis buffer (Cell Signaling Technology) with added protease inhibitor cocktail (Sigma-Aldrich). Extracts were centrifuged at 20,000×g for 10 min at 4°C and supernatant protein concentrations were determined using the DC Protein Assay (Bio-Rad Laboratories). Equal amounts of protein were separated by SDS polyacrylamide gel electrophoresis and transferred onto PVDF membranes. After blocking for 1 h with 5% non-fat dry milk in Tris-buffered saline (TBS), the membranes were incubated overnight at 4°C with primary antibodies that were diluted 1000-fold in 5% non-fat milk-TBS. Following primary antibody incubation, the membranes were incubated with the appropriate HRP-conjugated secondary antibody. Immunoreactive bands were detected using enhanced chemiluminescent substrate or SuperSignal West Femto chemiluminescent substrate and CL-XPosure film (Thermo Fisher, Ottawa, ON). Membranes were stripped with stripping buffer (50 mM Tris-HCl pH 7.6, 10 mM β-mercaptoethanol, and 1% SDS) at 50°C for 30 minutes and reprobed with anti-α-tubulin, anti-ERK1/2, anti-AKT or anti-p38 MAPK as loading controls. Immunoreactive band intensities were quantified by densitometry using Scion Image software (Scion Corp, Frederick, MD) and normalized to those of the relevant loading control.

### Small interfering RNA (siRNA) transfection and E-cadherin overexpression

To knock down endogenous SMAD2, SMAD3, SMAD4 and SNAIL, forty percent confluent cells were transfected for 48 h with 20 nM ON-TARGET*plus* SMART pool siRNA targeting human SNAIL, SMAD2, SMAD3 and SMAD4 (Dharmacon, Lafayette, CO) using Lipofectamine RNAiMAX (Invitrogen, Life Technologies). ON-TARGET*plus* Non-targeting pool siRNA (Dharmacon) was used as the control.

To overexpress E-cadherin, eighty percent confluent cells were transfected for 48 h with 1 μg empty vector (pcDNA3.1, Invitrogen, Life Technologies) or vector encoding full-length human E-cadherin (plasmid #45769, Addgene, Cambridge, MA) using Lipofectamine LTX (Life Technologies).

### Statistical analysis

Results are presented as the mean ± SEM of at least three independent experiments. For experiments involving only two groups, results were analyzed by Two-Sample t-test assuming unequal variances using Excel. Multiple group comparisons were analyzed by one-way ANOVA followed by Student-Newman-Keuls test using PRISM software (GraphPad Software). Means were considered significantly different if P < 0.05 and are indicated by different letters.

## SUPPLEMENTARY FIGURES


