# Supplementary material for: Activin B promotes endometrial cancer cell migration by down-regulating E-cadherin via SMAD-independent MEK-ERK1/2-SNAIL signaling
Source: Oncotarget. 2016 May 19;7(26):40060–72. doi: 10.18632/oncotarget.9483 (PMC5129992; doi:10.18632/oncotarget.9483)
Supplement: Supplementary file 1 [file oncotarget-07-40060-s001.pdf]

Activin B promotes endometrial cancer cell migration by down-regulating E-cadherin via SMAD-independent MEK-ERK1/2-SNAIL signaling

SUPPLEMENTARY FIGURES

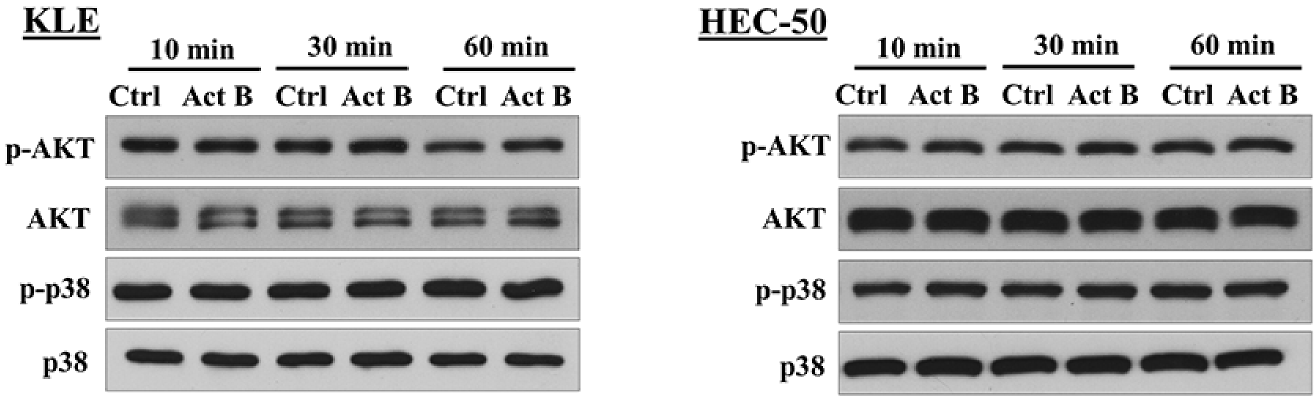

Supplementary Figure S1: Effects of activin B on AKT and p38 MAPK phosphorylation in endometrial cancer cells. KLE and HEC-50 cells were treated without (Ctrl) or with 50 ng/mL activin B (Act B) for 10, 30 or 60 min and Western blot was used to examine the levels of phosphorylated AKT (p-AKT) and p38 MAPK (p-p38) in relation to their total levels (AKT and p38, respectively).

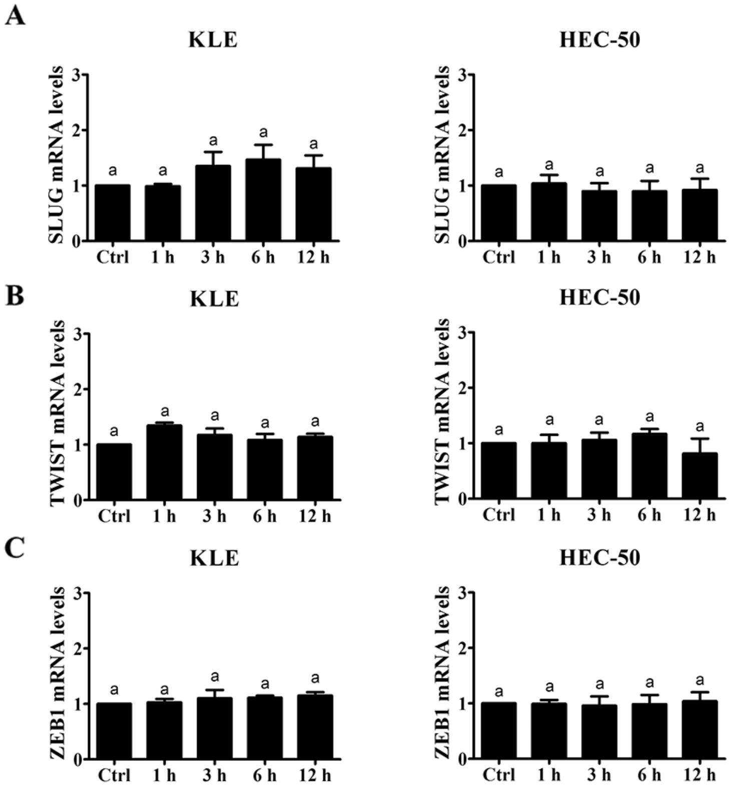

Supplementary Figure S2: Activin B does not alter the mRNA levels of SLUG, TWIST and ZEB1. KLE and HEC-50 cells were treated for varying times without (Ctrl; time-matched controls displayed as single bar) or with 50 ng/mL activin B and SLUG **A.** TWIST **B.** and ZEB1 **C.** mRNA levels were examined by RT-qPCR. Results are expressed as the mean ± SEM of at least three independent experiments and there were no significant differences between any of the groups.
